# Molecular docking analysis of netropsin and novobiocin with the viral protein targets HABD, MTD and RCD

**DOI:** 10.6026/97320630015233

**Published:** 2019-03-30

**Authors:** Afaf S Alwabli, Sana G Alattas, Alawiah M Alhebshi, Nidal M Zabermawi, Naser Alkenani, Khalid Al ghmady, Ishtiaq Qadri

**Affiliations:** 1 Department of Biological Sciences, Faculty of Science, King Abdulaziz University, Jeddah 21589, Saudi Arabia

**Keywords:** Dengue, therapeutics, helicase ATP binding domain, methyl transferase, RNA catalytic domain

## Abstract

Dengue, West Nile and Zika virus belongs to the family flaviviridae and genus flavivirus. It is of interest to design and develop inhibitors with improved activity against these diseases. We used the helicases target to screen for potential inhibitors against these viruses using molecular docking analysis. NS3 helicases of flavivirus family of viruses such as Dengue, West Nile and Zika are prime targets for drug development. The computer aided molecular docking analysis of netropsin and novobiocin with the viral protein targets HABD, MTD and RCD is reported for further consideration.

## Background

Dengue infection is a mosquito-borne human pathogen affecting by and large in tropical regions where 3.9 billion individuals live with around 100 million sicknesses showing clinical symptoms every year 1. Transmission of the disease occurs through mosquito bite of the Aedes class (for the most part Aedesaegypti and A. albopictus) 2. These mosquito vectors are commonly found in the middle of tropical regions 3. Most contamination is either asymptomatic or it causes smooth reactions (fever, joint agony or rashes) for a couple of days. However, available data on dengue hemorrhagic fever and dengue stun disorder raise to 20,000 cases in recent years 4. The dengue contamination has a spot with the flavi viruses family in addition to West Nile infection (WNV), dengue infection and the rising Zika infection (ZIKV) 5. Moreover, arbo viruses and Zika disease are also found to erupt lately in these regions 6. Zika virus contaminates are found in South America, Central America, and the Caribbean in addition to arbo viruses in the Western Hemisphere during the last two decades 7, 8. Without doubt, Zika diseases seeks attention after West Nile infection, dengue, which appeared in 1999 and chikungunya which appeared in 2013 8. The Zika contamination has a spot with the assortment flavivirus and is generally vectored by Aedes mosquitoes 9 found over the continents 10-12.

Helicases are omnipresent engine proteins that catalyze the unwinding of double-stranded DNA or RNA by ATP hydrolysis. They move along a nucleic acid phospho-di-ester spine and separate the complementary strands by utilizing energy got from ATP hydrolysis. 13 RNA helicases are important particles for RNA metabolic procedures, for example, ribosome biogenesis, joining and interpretation. It has been demonstrated that the helicases are connected to different segments of the macromolecular machines and they have critical role in numerous processes 14. Hence, the consideration of helicases as potential viral drug targets is realized.

The viral proteome is an extended polypeptide chain in the ER. It is further known that NS2A, NS2B, NS4A, and NS4B are structural layer proteins essential for viral replication through possible protein-protein and protein-lipid associations. The functions played by NS1, NS2A and NS4A in viral replication are complex to understand 15. NS1 has different oligomerization states dependent on its glycosylation status 16-18. The methyltransferase is in charge of topping the incipient genomic RNA by successively utilizing S-adenosyl methionine as the methyl donor. This is done by consecutive methylation on the N7 molecule with the top guanine and the 2'O particle of the ribose in the main adenine 19-20. Defect in capping decrease viral multiplication to cause infection that are gradually subtle to the natural resistant reaction as they stimulate higher interferon (IFN) flagging and immunizer reactions 21. Moreover, the positive sense of viral RNA is discharged in the cytoplasm during infection. NS5 protein initially deciphers it as a negative sense strand before utilizing the negative strand (with regards to a dsRNA middle of the road) to organize excess of positive sense RNA. The viral mRNA is then used to express the poly protein by host cell ribosomes. It is also known that a hetero-duplex framed by a DNA format and an RNA primer is formed 22. This component is available in the DENV NS5 full-length protein whose preference for dsDNA is like ssRNA 23. In the context of these available data it is of interest to screen HABD (helicase ATP binding domain), MTD (methyl transferase domain) and RCD (RNA catalytic domain) for potential inhibitors as drug candidates.

## Methodology

### Multiple sequence alignment (MSA) of the conserved domain:

The conserved domain sequences of dengue virus, zika virus and Japanese encephalitis virus were retrieved from the genome database in NCBI and BLASTp analysis was completed. The FASTA formats of the retrieved sequences were used for further analysis. Multiple sequence alignment (MSA) of Helicase ATP binding domain (201 amino acid residues), Methyl transferase domain (262 amino acid residues) and RNA Catalytic domain (149 amino acid residues) was completed using ClustalW3 and Clustal Omega. Various domains were manually assigned and confirmed by using Pfam, Prosite, SMART, PANTHER and InterProScan.

### Three-dimensional structure prediction by I-TASSER:

The protein sequence (201) of Helicase ATP binding domain (HABD), the protein sequence (262) of Methyl transferase domain (MTD) and the protein sequence (149) of RNA Catalytic domain (RCD) were downloaded from the Swiss Pro database. The threedimensional model was produced utilizing the I-TASSER server which creates a 3D model of inquiry arrangement by different threading arrangements and iterative necessary gathering reenactment 24. We used this server because its accessibility, composite methodology of displaying an execution in CASP rivalry. I-TASSER technique incorporates general strides of threading, and auxiliary get together, display determination, refinement, and structure-based comments 25. An optional structure was developed by PSIPRED 26 utilizing the structure library LOMETS 27. Z-score checked the nature of the formal arrangement followed by threading arrangements 24 using Monte Carlo simulation 28. The reenactment incorporates Ca/side chain connection, H-securities, hydrophobicity, spatial controls from threading template 27 and arrangement based on contact expectations from SVMSEQ 29. The adaptations produced amid the refinement reenactment process were bunched by SPICKER 30. Furthermore, the normal of three-dimensional directions of all the grouped structure was determined to acquire bunch centroids. In the refinement process, the chosen bunch centroids were again used to perform further reenactment, which evacuates steric conflicts to refine the topology of the group centroids. The known structures in PDB were recognized by TM-adjust 31. The final structural models were generated by REMO 32 in which group centroids of second-round reenactment were utilized. The useful analogs were ranked based on TM-score, RMSD, arrangement character, and the inclusion of the structural arrangement. The nature of the model was dictated by C-score (certainty score), which is -5 to 2. It depends on threading arrangement and the combination of auxiliary for refinement reenactments.

### Preparation of ligands and protein targets:

The 3D structures of HABD, MTD and RCD were built using ITASSER. The hydrogen atoms having polar nature were then included. The buildup structures less in numbers were removed and the fragmented side chains were later replaced by Auto Dock Tools (ADT) version 1.5.6 downloaded from the Scripps Research Institute. Further, particles having Gasteiger charges were included and the non-polar hydrogen iotas were added to the protein structure. The built structures were then stored in PDBQT format in ADT 33. 3D structures of netropsin and novobiocin were drawn using ChemBioDraw Office 12.0. The 3D co-ordinates of the ligands was then created and stored in PDBQT format using ADT 33.

### Receptor grid formation:

Networks predetermine matrix maps of restricting energies in various particle types (for example, hydrogen holding oxygen, carbons, and aliphatic carbons in a macromolecule (for example a RNA/DNA, protein)) before docking 34. These network maps are then utilized in AutoDock 4.2 docking computations to characterize the absolute restricting vitality for a ligand with a macromolecule 33. Network mapping computes the vital parameters over the protein nuclear information and determines the directions of the HABD, MTD, and RCD for docking. Likewise, lattice mapping manages an appropriate surface topology for the iotas of mixes for association with the HABD, MTD and RCD dynamic sites. Network mapping is a necessity to guide netropsin, and novobiocin mixes to search for their locale for binding with the HABD, MTD and RCD dynamic sites.

The network measurements for the HABD protein was 58 x 48 x 52 matrix focused with separating 1.00 Å between the framework focused on the ligand for protein (59.686, 70.660 and 46.318 directions). The framework measurements for MTD protein was 48 x 52 x 58 matrix focused with dispersing 1.00 Å between the network focuses; however fixated on the ligand for protein (61.363, 69.710 and 54.870 co-ordinates). The lattice measurements for malate synthase protein was 52 x 52 x 55 framework focused with dividing 1.00 Å between the framework focuses yet fixated on the ligand for protein (60.820, 65.487 and 70.722 directions). The network was made for selecting promising cooperation for docking between ligands and targets 35.

### Molecular docking:

AutoDock 4.2 with standard parameters was used to dock the netropsin, and novobiocin mixes into the dynamic sites of HABD, MTD, and RCD 33, 36. The energy calculation was done using the Lamarckian hereditary estimation (LGA). The adaptations with the most significant free restricting vitality were chosen for dissecting the connections between the receptor and ligands using PyMOL and Discovery studio 33.

## Results and Discussion

### Sequence analysis and domain organization:

**Figure 1:**
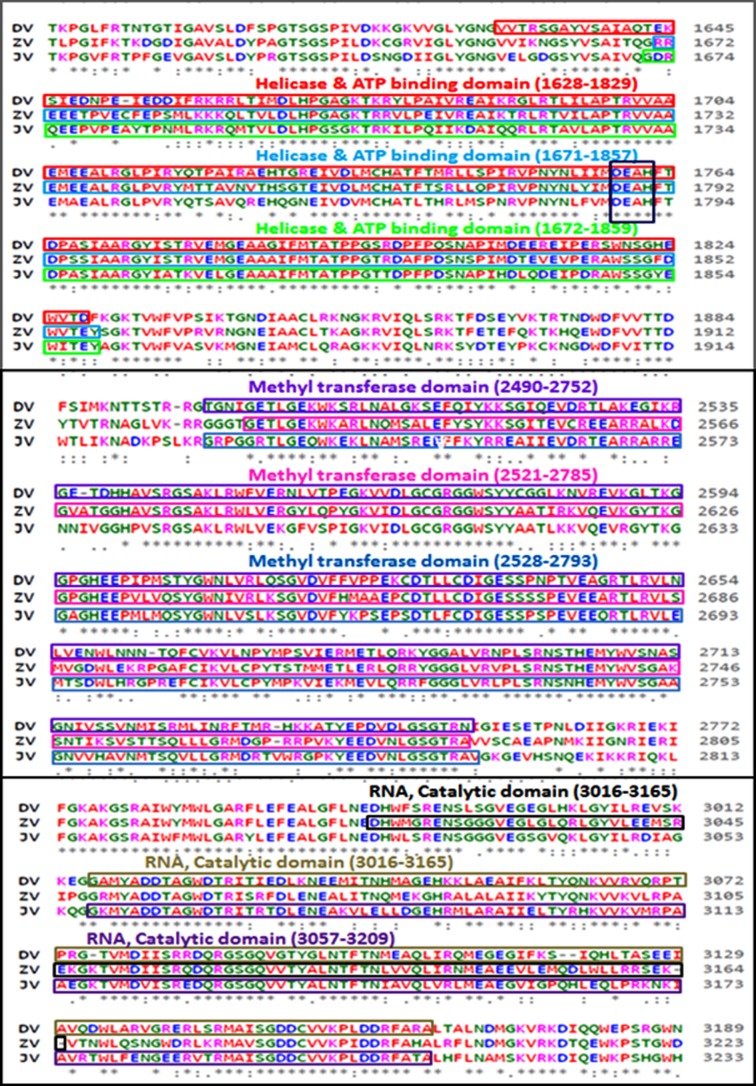
Multiple sequence alignment (MSA) of conserved domain amino acid sequence of dengue virus, zika virus and japanese encephalitis virus. MSA was done using the CLUSTALW2 program (http://www.ebi.ac.uk).

**Figure 2:**
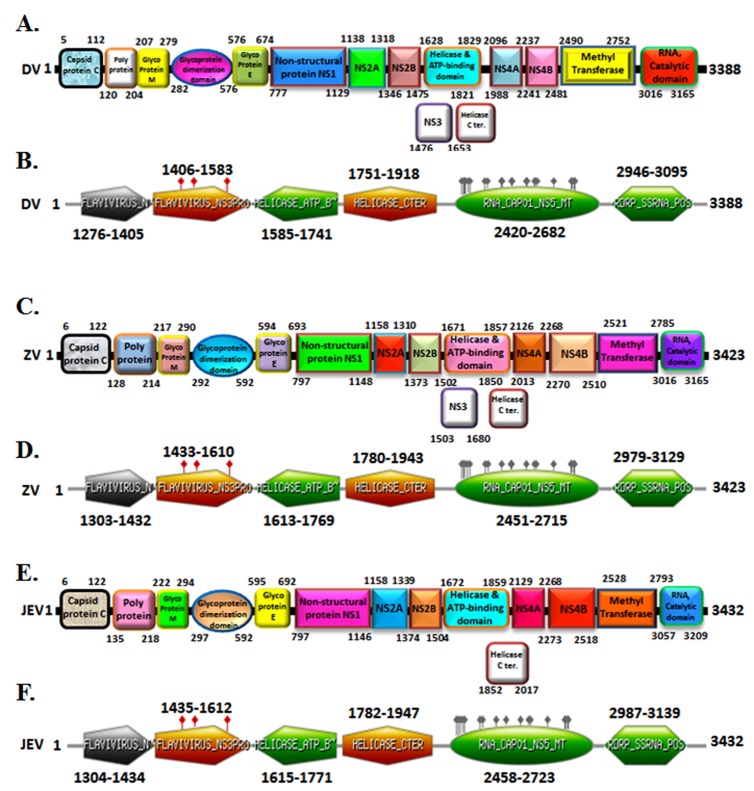
Domain organization of Dengue virus, Zika virus and Japanese encephalitis is shown. The conserved sequences of each domain are written inside the boxes. The text written in a box of various conserved domains and the numbers refer to the amino acids spanning the various domains.

An analysis of amino acid alignment sequence of three unique domain that is Helicase ATP binding domain (201 amino acid), Methyl transferase domain (262 amino acid) and RNA Catalytic domain (149 amino acid) shown in Figure 1 was done using the Clustal Omega program. The InterProScan examination demonstrates that Dengue, Zika infection and Japanese Encephalitis viruses have conserved motifs (Figure 2 A-F). The comparative study provided useful insights on conserved domains, which are present in all three viruses. This is useful to study targets for antiviral drug design.

### Three-dimensional structure models:

**Figure 3:**
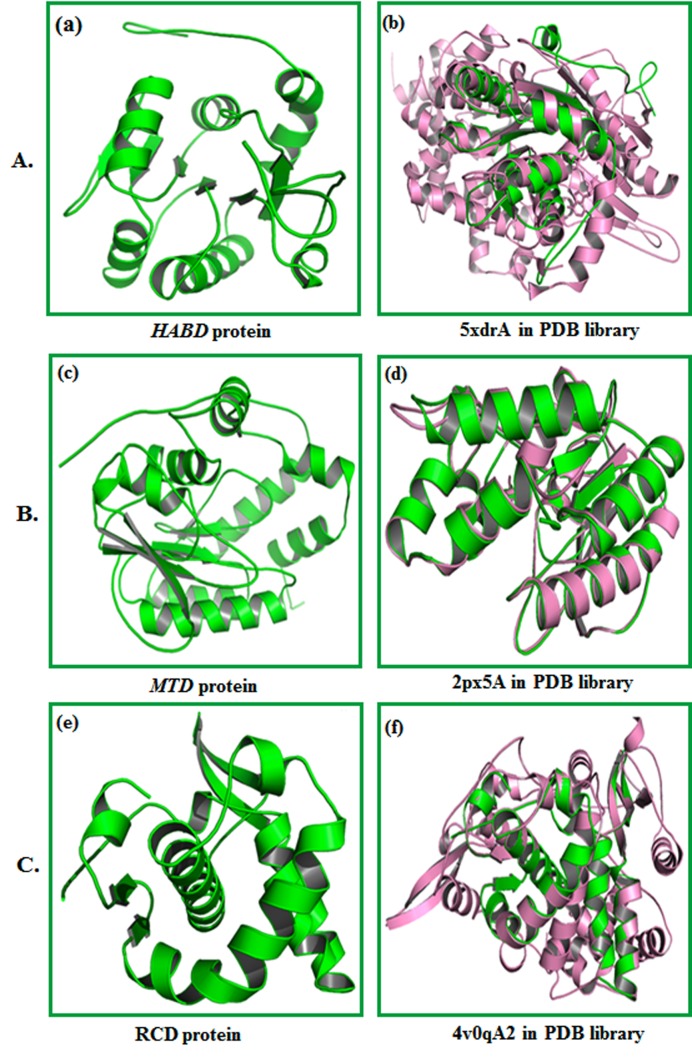
(A) (a) Three-dimensional structure of HABD protein anticipated by I-TASSER. (b) Alignment of question protein (Green) with auxiliary simple (Pink) 5xdrA in PDB library. (B) (c) Threedimensional structure of MTD protein developed by I-TASSER is shown. (d) Alignment of question protein (green) with basic simple (pink) 2px5A in PDB library. (C) (e) Three-dimensional structure of RCD protein anticipated by I-TASSER.(f)Alignment of inquiry protein (green) with auxiliary simple (pink) 4v0qA2 in PDB library.

The molecular model of HABD, MTD, and RCD proteins was developed using I-TASSER. The models obtained from server incorporate supporting structure with certainty score (0 to 9), anticipated dissolvable openness. We obtained five models with C-score, top ten formats from PDB used in the arrangement; top ten PDB basic analogs, useful analogs of protein, and restricting site deposits. The HABD model (Figure 3A) was chosen with Cscore 0.92, TM-score 0.60 ± 0.14 and RMSD 7.3 Å 4.2 Å. MTD (Figure 3B) was selected as the best-expected model with C-score 1.17, TM-score 0.87±0.07, and RMSD 3.6 ± 2.5 Å. MTD (Figure 3 C) was chosen as the best model with C-score 0.99, TM-score 0.85 ± 0.08, and RMSD 2.8 ± 2.1 Å. C-score with higher esteem mirror a model for better quality 18. Standardized Zscore commonly evaluates threading. A normalized Z-score > 1 esteem mirror a specific arrangement. TM-adjust distinguished 5xdrA, 2px5A and 4v0qA2 in PDB library as the best scoring models from I-TASSER with a TM-score of 0.792, 0.979 and 0.979, respectively.

### Molecular docking studies:

**Figure 4:**
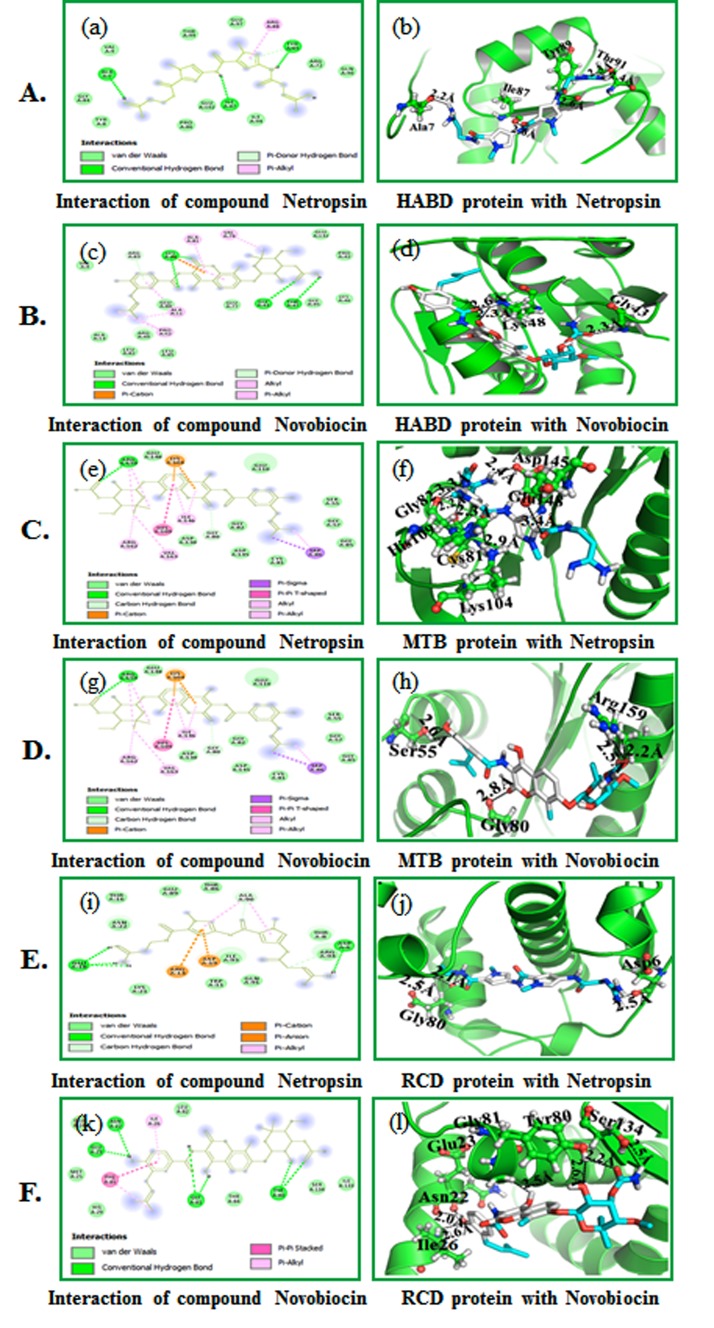
Molecular docking of compounds with HABD: (A) (a) 2D schematic diagram showing interactions of compound netropsin. (b) Cartoon view of HABD protein with compound netropsin. (B) (c) 2D schematic diagram showing interactions of compound novobiocin. (d) Cartoon view of HABD protein with compound novobiocin. Residues involved in hydrogen bonding, van der Waals interactions, carbon hydrogen and Pi-alkyl are represented in different color indicated in the inset. (C) (e) 2D schematic outline appearing of compound netropsin. (f) Cartoon perspective on MTD protein with compound netropsin. (D) (g) 2D schematic outline appearing of compound novobiocin. (h) Cartoon perspective on MTD protein with compound novobiocin. Deposits engaged with hydrogen holding, van der Waals (vdW) cooperation, carbon hydrogen and Pi-alkyl are spoken to in various shading shown in the inset. (E) (i) 2D schematic graph appearing of compound netropsin. (j) Cartoon perspective on RCD protein with compound netropsin. (F) (k) 2D schematic graph appearing of compound novobiocin. (l) Cartoon perspective on RCD protein with compound novobiocin. Deposits engaged with hydrogen holding, van der Waals (vdW) cooperation, carbon hydrogen and pi-alkyl are spoken to in various shading demonstrated in the inset.

Auto docking 4.2 was used to set up the coupling capacity of the netropsin, and novobiocin mixes with HABD, MTD, and RCD for docking interactions. We examined the interaction of HABD, MTD and RCD with the inhibitors using PyMOL. Results show that the compound netropsin is compatible with the dynamic site of HABD, MTD and RCD with least restricting vitality (ΔG) -6.7 kcal/mol, (ΔG) -7.3 kcal/mol and -6.3 kcal/mol. The compound novobiocin is pleasantly limited into the dynamic site of HABD, MTD and RCD with least restricting vitality (ΔG) -7.5 kcal/mol, (ΔG) - 8.9 kcal/mol and -7.8 kcal/mol. The compound netropsin forms four hydrogen bonds - one each with HABD buildup (Ala7; 2.2 Å, Ile87; 2.6 Å, Tyr89; 2.6 Å and Thr91; Ile87; 2.4 and 2.3 Å) (Figure 4A (b).

The compound netropsin is compatible inside the HABD binding site by connecting with different deposits appeared in Figure 4C (e). The compound novobiocin forms three hydrogen bonds with the HABD buildup (Gly43; 2.3 Å and Lys48; 2.3 and 2.6 Å) as shown in Figure 4D (h). The compound novobiocin likewise communicates with the HABD restricting site by associating with different deposits appeared in Figure 4D (g). The compound netropsin through six hydrogen bonds with MTD (Cys81; 2.3 Å, Gly82; 2.2 Å, Lys104; 2.9 Å, His109; 3.3 Å, Glu148; 3.4 Å and Asp145; 2.4 Å) as shown (Figure 4E (j). It communicates with the MTD binding site by interfacing with different deposits appeared in Figure 4E (i). Novobiocin associated well through three hydrogen bonds MTD buildup (Ser55; 2.6 Å, Gly80; 2.8 Åand Arg159; 2.2 and 2.5 Å,) appeared in Figure 4F (l). The compound novobiocin likewise associates with the MTD restricting site by collaborating with different build ups appeared in Figure 4F (k). Compound netropsin showed three hydrogen bonds with RCD build up (Asp6; 2.5 Å and Gly80; 2.1 and 2.5 Å) (Figure 4F (l). The compound netropsin likewise accepts good interaction inside the RCD restricting site by interfacing with different buildups as shown in Figure 4E (i). Novobiocin collaborated through six hydrogen securities RCD build up (Asn22; 2.0 Å, Glu23; 2.0 Å, Ile26; 2.6 Å, Tyr80; 2.2 and 2.6 Å, Gly81; 2.5 Å and Ser134; 2.5 Å) as shown in Figure 4F (l). The compound novobiocin likewise collaborates with the RCD restricting site by associating with different deposits as shown in Figure 4F (k).

## Conclusion

We report the molecular docking analysis of netropsin and novobiocin against the helicases targets of Dengue, West Nile and Zika viruses. The analysis shows that netropsin and novobiocin bind to viral targets HABD, MTD and RCD with high binding ability for further in vitro and in vivo studies.

## Conflict of Interest

Authors declare no conflict of interest.

## References

[1] Wilder-Smith A (2016). The Journal of infectious diseases.

[2] Gratz N (2004). Medical and veterinary entomology.

[3] Machado CM (2009). Revista do Instituto de Medicina Tropical deSao Paulo.

[4] Kuno G (1997). Dengue and dengue hemorrhagic fever. New York: CABInternational.

[5] Samarasekera U, Triunfol M (2016). The Lancet.

[6] Benelli G, Mehlhorn H (2016). Parasitol Res.

[7] Attar N (2016). Nature Reviews Microbiology.

[8] Fauci AS, Morens DM (2016). New England Journal of Medicine.

[9] Marcondes CB, Ximenes MdFFd (2016). Revista da SociedadeBrasileira de Medicina Tropical.

[10] Mehlhorn H (2012). Arthropods as vectors of emerging diseasesSpringer Science and Business Media..

[11] Becker N (2012). Exotic mosquitoes conquer the world,.

[12] Melaun C (2015). Parasitology Research.

[13] Cordin O (2006). Gene.

[14] Bae S (2010). Nature Nanotechnology.

[15] Yu LK (2013). Virology.

[16] Rastogi MN (2016). Virology Journal.

[17] Somnuke P (2011). Virology.

[18] Avirutnan P (2007). PLoS Pathogens.

[19] Zhao Y (2015). Proceedings of the National Academy of Sciences.

[20] Issur M (2009). RNA.

[21] Decroly E (2012). .Nature Reviews Microbiology.

[22] Lu G (2017). Antimicrobial Agents and Chemotherapy.

[23] Szymanski MR (2011). J Biol Chem.

[24] Wu S (2007). BMC Biology.

[25] Roy AA (2010). Nature Protocols.

[26] Jones DT (1999). Journal of Molecular Biology.

[27] Wu S, Zhang Y (2007). Nucleic Acids Research.

[28] Zhang Y (2002). Proteins: Structure, Function and Bioinformatics.

[29] Wu S, Zhang Y (2008). Bioinformatics.

[30] Zhang Y, Skolnick J (2004). Journal of computational chemistry.

[31] Zhang Y, Skolnick J (2005). Nucleic Acids Research.

[32] Li Y, Zhang Y (2009). Proteins: Structure, Function, and Bioinformatics.

[33] Ahmad K (2017). Letters in Drug Design and Discovery.

[34] Gill BS, Kumar S (2015). Medicinal Chemistry Research.

[35] Repasky MP (2007). Current Protocols in Bioinformatics.

[36] Morris GM (1998). J. Comput. Chem..

